# Improved performance and design of a low-cost laparoscope to enable laparoscopic surgery in low-income countries

**DOI:** 10.1117/1.BIOS.2.2.022302

**Published:** 2025-02-03

**Authors:** Anne Christine Barnes, Michele L. Kaluzienski, Jason Chen, Tri T. Quang, Talya Simcox, Paula Kworekwa, Rebecca Kaaya, Robert Ssekitoleko, Kevin Aroom, John Rzasa, Tamara Fitzgerald, Jenna L. Mueller

**Affiliations:** a University of Maryland, Department of Bioengineering, College Park, Maryland, United States; b Makerere University, Biomedical Engineering Unit, Department of Physiology, School of Biomedical Sciences, College of Health Sciences, Uganda; c University of Maryland, Robert E. Fischell Institute for Biomedical Devices, College Park, Maryland, United States; d Duke University Medical Center, Department of Surgery, Durham, North Carolina, United States; e Duke University, Duke Global Health Institute, Durham, North Carolina, United States; f University of Maryland School of Medicine, Department of OB-GYN and Reproductive Science, Baltimore, Maryland, United States; g University of Maryland School of Medicine, Marlene and Stewart Greenebaum Cancer Center, Baltimore, Maryland, United States

**Keywords:** laparoscopic surgery, biomedical optics, low- and middle-income countries, global surgery, global engineering, frugal medical device

## Abstract

**Significance:**

Laparoscopy has become the standard of care for surgery in the chest and abdomen but is typically unavailable in low- and middle-income countries (LMICs). This inaccessibility is partly due to a high initial equipment purchase price, ongoing maintenance costs, unreliable electricity, shortage of biomedical technicians, and limited sterilization facilities.

**Aim:**

To address these challenges, a low-cost, durable, reusable laparoscopic system (KeyScope) was designed for use in LMICs.

**Approach:**

Through an iterative human-centered design approach, the performance of the KeyScope was optimized by comparing standard image quality metrics to a commercially available standard-of-care (SOC) laparoscope.

**Results:**

The latest version of the KeyScope has comparable or better resolving power, lens distortion, field of view, depth of field, and color reproduction accuracy to a SOC laparoscope (Precision Ideal Eyes HD Laparoscope, Stryker) at working distances commonly used during laparoscopic surgery (3 to 13 cm). Interference from electrocautery was eliminated by shielding the camera ground from the housing ground. Finally, the entire KeyScope is equipped for production and implementation in sub-Saharan Africa, as the device can be submerged in Cidex and can be easily assembled in Uganda in under 1 h.

**Conclusion:**

These results suggest that the KeyScope has achieved the performance criteria needed for surgical care in LMICs.

Statement of Discovery This work utilizes miniaturized complementary metal-oxide semiconductors and light emitting diodes to create a low-cost laparoscope designed for use in low and middle-income countries. This technology will cultivate increased access to laparoscopic surgeries in regions with poor healthcare accessibility.

## 1 Introduction

Laparoscopic surgery significantly reduces post-surgical morbidity compared to open surgery due to the avoidance of large incisions.^1^ Studies comparing postoperative complications between open surgery and laparoscopy consistently demonstrate reduced mortality, surgical site infections, hospital stay, and pneumonia.^2^^,^^3^ These advantages have been observed across surgical specialties, which has led to the widespread adoption of laparoscopic surgery in high-income countries.^1^^,^^4^^,^^5^

Although laparoscopic surgery has clear advantages over open surgery, it is typically unavailable in low- and middle-income countries (LMICs), except in limited tertiary centers.^1^ To further understand the challenges of laparoscopic surgery in LMICs, our team previously conducted interviews with LMIC surgeons.^6^ We found that not only is the initial cost of standard-of-care (SOC) units prohibitively expensive (>$130,000 per operating room), but the equipment is fragile and frequently requires repair. Laparoscopes are also composed of several components which must be sterilized and reassembled after each use.^7^ If one of these parts is lost or broken, it is difficult to replace or repair. In high-income countries, hospital systems maintain expensive service contracts with medical device companies, but these service contracts are typically not affordable in LMICs. Local repair of broken devices is often not possible as there are scarce biomedical technicians and repair manuals are proprietary.^8^^–^^10^ In addition, SOC laparoscopes require a continuous power supply and many countries have frequent power outages.^11^ Laparoscopic components are typically sterilized in large autoclaves or via ethylene oxide gas sterilization, both of which require a significant amount of equipment and hospital infrastructure that are often not available in LMICs.^7^ Thus, there is a need to design an affordable laparoscope that meets the needs of LMICs.^6^^,^^9^^,^^10^^,^^12^

SOC laparoscopes typically include (1) a laparoscopic camera unit that contains a microelectronic video camera (utilizing a high-resolution CCD), (2) a high-intensity light source, such as a Xenon arc lamp, which is coupled to a fiber optic light guide cable, (3) rigid optics within the laparoscopic probe (to transmit light through the body cavity to the camera), and (4) a digital video capture unit to record, process, and visualize video from the camera on a monitor.^13^ To address the needs of LMICs, our goal was to develop an affordable, robust, single-unit laparoscopic system, called the KeyScope, that can operate in power outages and is compatible with submersion sterilization methods. Rather than using fiber optic cables, the KeyScope contains a ring of light-emitting diodes (LEDs) and a CMOS detector that is moved to the tip of the device.^14^ This modification significantly decreases the cost, and there are no separate parts to be lost during sterilization. The KeyScope connects to a laptop computer via a USB, which provides power to the device and displays the image on the laptop. This eliminates the need for expensive monitors and enables surgeons to continue using the device for approximately two additional hours during power outages or longer with additional laptop battery support. The KeyScope is waterproof and can be sterilized by chemical immersion. It was designed to be manufactured in Uganda, which increases local biomedical device capacity, and provides avenues for local maintenance and repair. The KeyScope would benefit both hospitals that currently do not have any access to laparoscopic equipment (and therefore open procedures are performed) and hospitals that may have access to SOC laparoscopic equipment but find that the equipment is difficult to maintain.

Although the initial design of the KeyScope demonstrates its feasibility, key areas for improvement were identified in previous work. Here, we describe how we further improved upon the KeyScope’s design through an iterative human-centered design approach, which emphasizes the end user’s needs. Human-centered design traditionally has three phases: hear, create, and deliver.^15^ In the hearing phase, designers learn more about the problem and hear what end users say about the latest prototype. In the creation phase, design changes are implemented to further address end users’ needs. In the delivery phase, the latest prototype is delivered to end users to obtain feedback about the design. Our end users included surgeons who have experience operating in LMICs and engineers who have experience developing, building, and implementing biomedical devices in LMICs. Specifically, surgeons performed studies on swine and provided feedback on the image quality and usability of the device. The prototypes were additionally shown to surgeons and engineers at stakeholder meetings in Uganda.

Through this iterative human-centered design approach, we improved upon several design aspects, including improving image resolution at longer working distances, increasing light output, and testing its performance *in vivo*. The first-generation of the KeyScope demonstrated the feasibility of using a CMOS sensor and ring of LEDs placed at the tip of the probe.^14^ Although it achieved comparable distortion, field of view, and color reproduction accuracy to a commercially available SOC laparoscope, the feature resolution of the first-generation KeyScope was only comparable to the SOC laparoscope at shorter working distances of 3 to 4 cm. As the working distance increased to 5 cm and beyond, the ability of the KeyScope to resolve features declined. Thus, in the second-generation design, we addressed limited feature resolution at longer working distances of ≥5  cm. We also addressed several issues with the form factor, or housing, of the design, which were identified by end users. This included changing the angle of the handle to improve ergonomics and developing a waterproofing strategy to enable submersion of the entire device in Cidex to comply with reprocessing techniques in LMICs.^7^ Next, in the third-generation design, we improved the light output by using a unique strobe approach in which LEDs are pulsed to increase the light intensity while simultaneously reducing heat production. The LEDs are pulsed in sync with the frame clock of the camera so that the LEDs always appear “on” to the surgeon viewing the video stream. Pilot manufacturing of the third-generation KeyScope was performed in a local makerspace in Uganda to assess manufacturing feasibility in an LMIC. This enabled the identification of steps to increase the efficiency and ease of the assembly process. Finally, to evaluate the KeyScope performance *in vivo*, the third-generation design was tested in swine, a commonly used model for laparoscopy.^16^ Through *in vivo* testing, it was observed that cautery tools occasionally interfered with the video stream from our device when the ground pad was inadequately adhered to the subject. Thus, in the fourth-generation design, the camera and LEDs were electrically isolated from the device’s metal housing. Each generation of the KeyScope was tested via a series of bench tests in a portable optical testing chamber to ensure image quality was preserved.^14^^,^^17^

## 2 Materials and Methods

### 2.1 Second-Generation KeyScope—Improvement of Resolution and Form Factor

To improve the resolution of the KeyScope at ≥5  cm working distances, we developed a customized camera module that includes a color CMOS camera, a ring of eight high-intensity white LEDs, and a lens with an F-number of 5.0, which improves the depth of field characteristics. The distance from the umbilicus to the outer edges of the abdominal cavity can be 12 cm;^18^ thus, the range of working distances needed for laparoscopy is estimated to be 3 to 13 cm. The second-generation KeyScope contained a 0.3 megapixel (MP) CMOS camera, whereas later versions (third and fourth-generations) contained a 2.0 MP CMOS camera that further improved the resolution. Camera modules were housed within a 5.5 mm outer diameter metal tube, which is compatible with standard trocar ports.

**Fig. 1:**
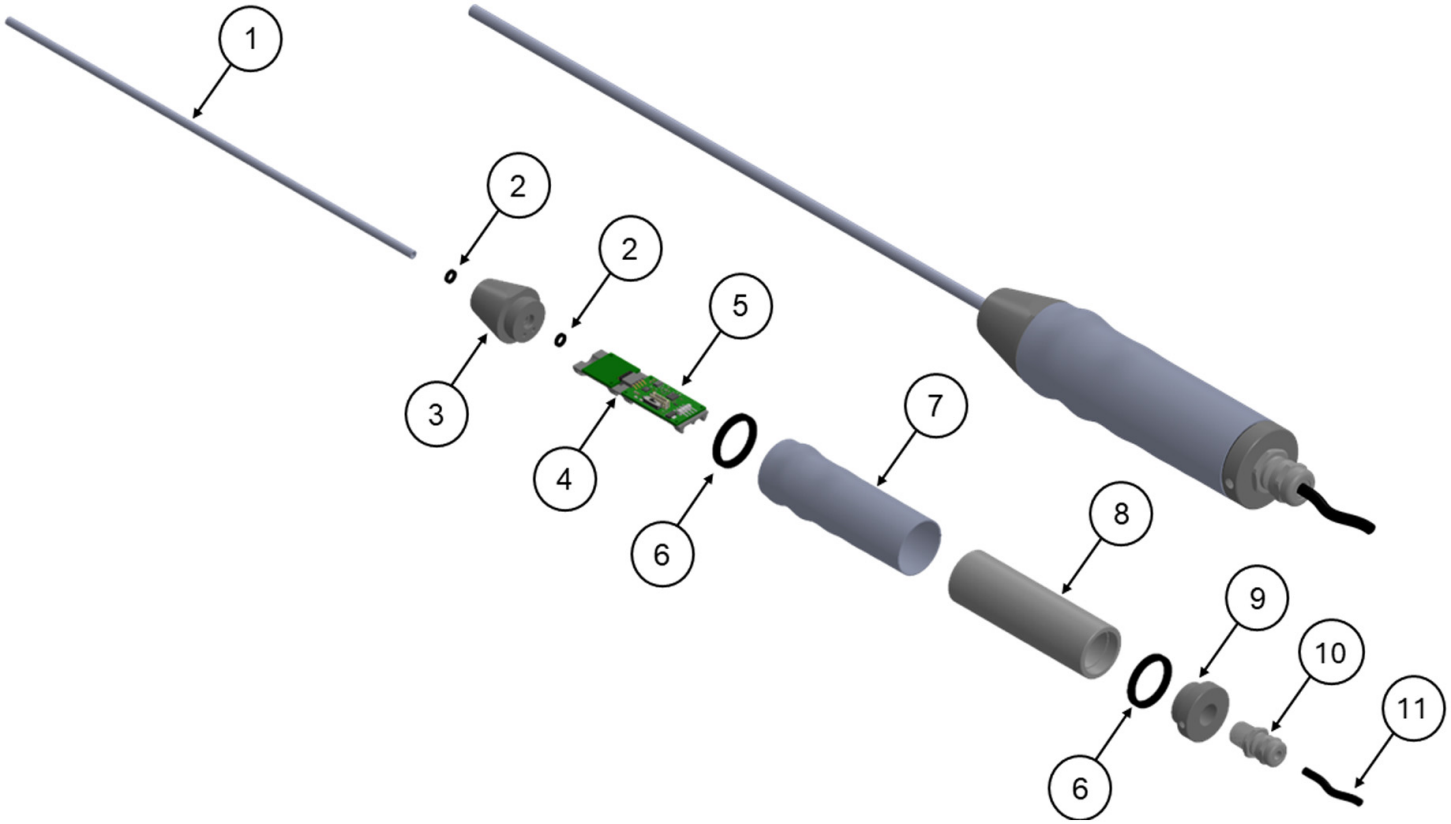
Rendering of KeyScope and exploded view of 12 individual components, including (1) camera module, (2) small O-rings, (3) top cap, (4) PCB sled, (5) custom PCB, (6) large O-rings, (7) hand grip, (8) large tube, (9) bottom cap, (10) strain relief, and (11) USB cable. Components 1 to 3 and 7 to 12 were incorporated into the second-generation KeyScope. Components 4 to 6 were incorporated into the third-generation KeyScope.

Several additional improvements were also addressed in the second-generation KeyScope. Specifically, end users suggested that the handle should be changed to a 0-deg angle relative to the probe (rather than the previous 135-deg angle). In addition, the previous 3D-printed handle had a rectangular-like design with squared-off edges. Surgeons preferred a rounded design with a handle grip for a more ergonomic feel. Finally, whereas the tip of the first-generation KeyScope was waterproofed to protect the camera module, the entire device needed to be submersed in Cidex OPA to be reprocessed between patients. To address these three issues, the handle was redesigned to contain modular components for the camera tube, handle grip, end caps, O-ring seals, cord grip, and cable connectors. Figure 1 shows an exploded view of the modular housing. The handle has a 3D-printed grip that surrounds the inner handle; this tube within a tube design maintains watertight protection while providing ergonomic benefits to the surgeon. To confirm that the design was watertight, a second-generation KeyScope was submerged in a tray of Cidex for 30 mins, then rinsed with deionized water (the method typically used by surgical nurses in Uganda) and left to air dry. Before and after submersion, images of the USAF 1951 resolution target were taken at a 3 cm working distance, and the illumination level was measured with a light meter (Sper Scientific, 840006) to ensure that camera and LED functionality were maintained as described in Ref. 14.

### 2.2 Third-Generation KeyScope—Improving Light Output and Manufacturability

Previous results indicated that lux values were less than a third of that generated by the SOC laparoscope.^14^ To increase the illumination in the third-generation KeyScope, the LEDs were pulsed at a high frequency, but low-duty cycle. The light strobe was synced with the frame clock of the camera, allowing for bright illumination while the camera is exposed, but resting the LEDs while the camera is off. A light meter (B&K Precision 615) was used to assess light intensity at a variety of working distances as described in Ref. 14. Because the light is pulsed in the third-generation KeyScope, the LEDs were briefly switched to DC mode, so that the light remained on during bench testing. To measure the temperature of the KeyScope in a simulated *in vivo* environment, the probe portion of the second-, third-, and fourth-generation KeyScopes was placed in an Erlenmeyer flask with 200 mL of water, which was heated to a temperature of 37°C to mimic *in vivo* temperature and humidity. A thermocouple was placed at the probe tip and the temperature was measured every minute for 1 h.

**Fig. 2:**
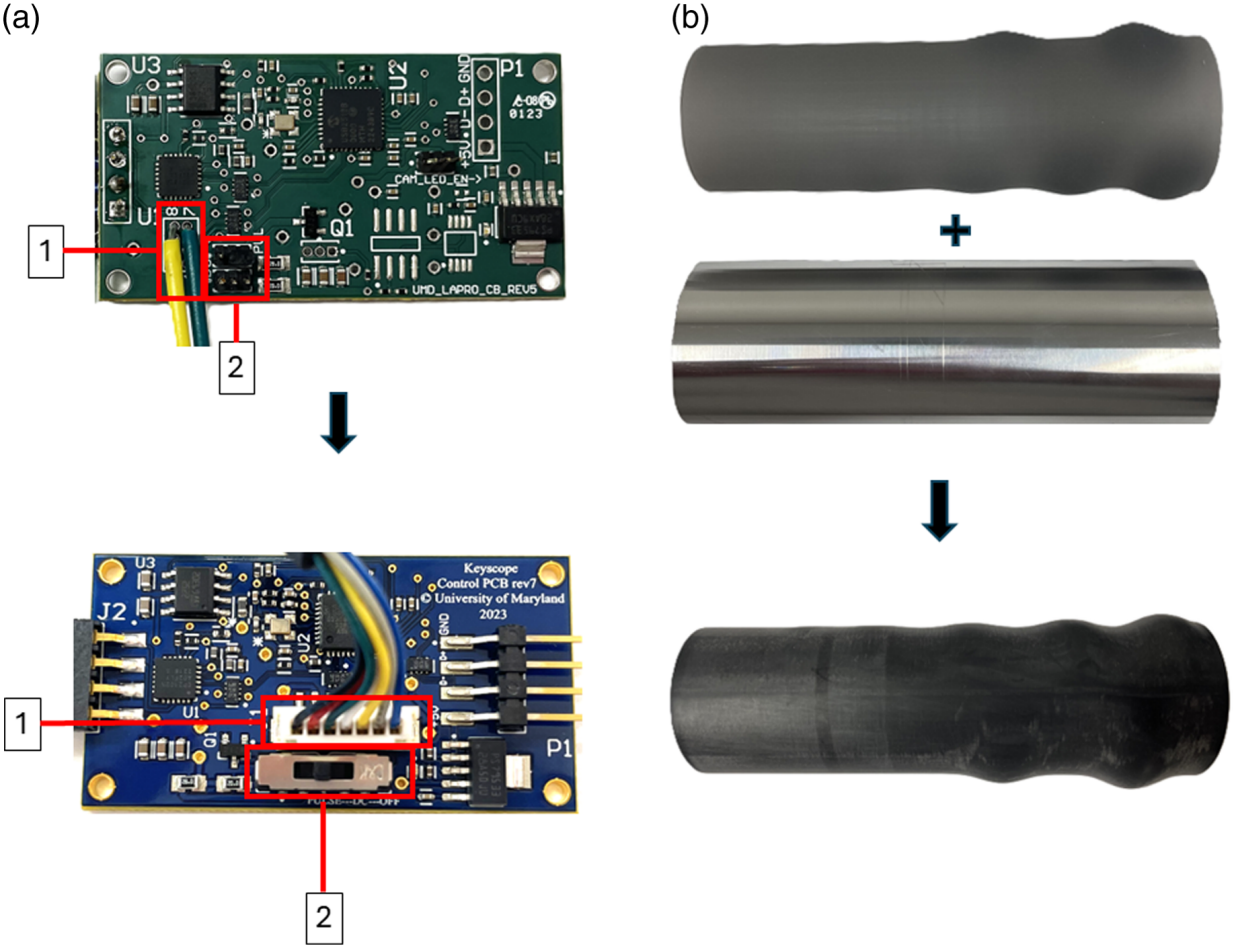
KeyScope design changes from third-generation to generation 3.2. (a) The top image shows the third-generation PCB, where 1 indicates where the camera wires were soldered into the board and 2 indicates the shunts used to change the LED settings. The bottom image displays the generation 3.2 PCB, where 1 indicates a plug for the camera wires in place of soldering and 2 indicates the use of a switch to change the LED settings, replacing the shunts in third-generation. (b) The top image shows the third-generation handle pieces, consisting of a separate 3D-printed grip and center tube to encapsulate the circuitry components. These two pieces were fitted together using a clamp. The bottom image shows the generation 3.2 handle piece, which is entirely 3D printed with the grip and center tube combined, which eliminates the clamping step.

The third-generation KeyScope was delivered to biomedical engineers in Uganda to determine if the device could be manufactured locally. Components and assembly instructions needed to build five devices were delivered to a renovated shipping container makerspace on Makerere University’s campus. Local engineers were trained to build the devices and then each step in the assembly process was timed. Steps included (1) probe and top cap assembly, (2) handle assembly, (3) circuit board assembly, and (4) USB cord wiring and final closure. Then, changes were made to the assembly process to decrease overall assembly time. Specifically, male and female connectors were added to the camera and printed circuit board, respectively, to reduce the time required for circuit board assembly (Fig. 2).

### 2.3 Fourth-Generation KeyScope—Addressing Interference from Cautery

The third-generation KeyScope was tested in pigs by our surgical colleagues (under Duke Institutional Animal Care and Use Committee Protocol #: A219-19-10). Occasional cautery interference was observed as suspension of the camera video feed, particularly when the grounding pad was poorly adhered to the animal. Thus, the KeyScope’s video feed was tested in different configurations with a cautery tool (SurgiStat II, Valleylab, MN, set to fulgurate 40): (1) cautery was held near the tip of the KeyScope, (2) cautery and KeyScope were held in direct contact with excised tissue on a grounding pad, and (3) cautery was held in direct contact with the KeyScope. To address interference from cautery, the camera and LED ground wires were electrically isolated in the fourth-generation KeyScope to shield the camera ground from the housing ground. With this arrangement, the high-energy shocks from the cautery tool were redirected away from the camera and to the chassis ground.

### 2.4 Image Quality Characterization of Second-, Third-, and Fourth-Generation KeyScopes

The image quality of each generation was characterized by imaging various targets as described previously.^14^ Briefly, targets included the USAF 1951 resolution target, SFRplus geometric distortion target, 5 to 15 depth of field gauge, and NIST-calibrated color checker target. The custom optical hardware setup was secured to an optical table. However, to facilitate testing of KeyScope performance in Uganda, a portable testing chamber was developed. The chamber was made out of foam walls with a black exterior and white interior, which were selected to block ambient light while enabling uniform illumination of optical targets. Diffuse white LEDs were placed at the top front of the chamber and the light sources from the laparoscopes were turned off to achieve consistent light exposure of the imaging targets. Five measures of each target were captured and compared with a SOC laparoscope (Precision Ideal Eyes HD Laparoscope, Stryker, MI). The resolution and depth of field target images were analyzed using ImageJ (National Institutes of Health, WI), and the geometric distortion target and NIST-calibrated color target were analyzed with Imatest (Imatest LLC, CO) as described previously in Ref. 14.

### 2.5 Statistical Analysis

Five images of each target were captured and analyzed for all experiments unless otherwise indicated. Wilcoxon rank sum, Kruskal-Wallis, and Dunn’s tests (non-parametric, two-tailed alpha = 0.95) were performed in MATLAB (MathWorks, Natick, Massachusetts, United States) to determine significant differences between the KeyScope and SOC laparoscopes. A significance level of p≤0.05 was considered to reject the null hypothesis.

## 3 Results

### 3.1 Resolution and Waterproof Testing

**Fig. 3:**
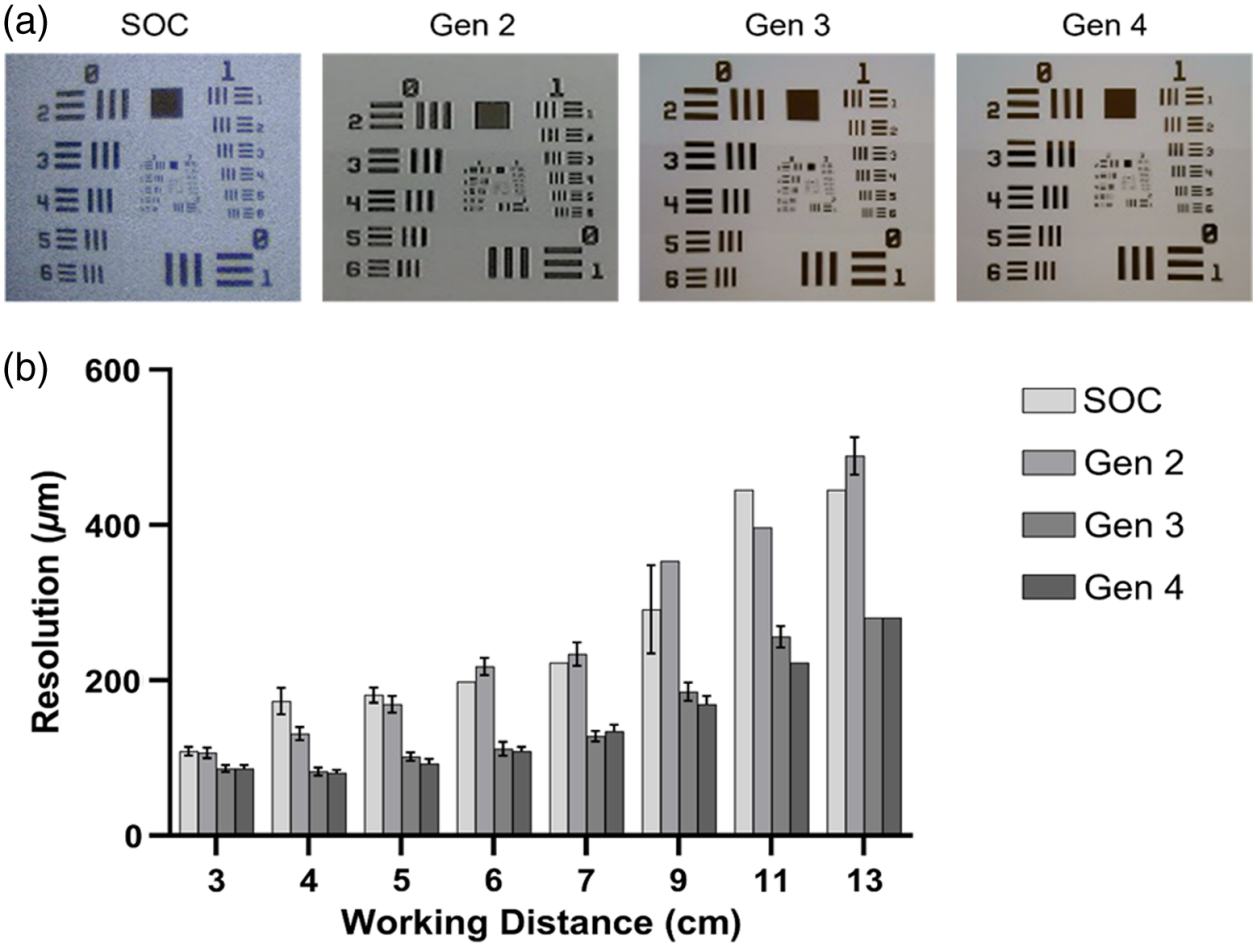
(a) Representative images of the USAF 1951 resolution target captured with an SOC laparoscope and second-, third-, and fourth-generation KeyScopes. (b) The limit of resolution was measured at a variety of working distances for the second-, third-, and fourth-generation KeyScopes and compared with a SOC laparoscope. Error bars indicate standard deviation, and each group had a sample size of n=5. All comparisons are significant with p<0.05, except for SOC versus Gen 2 at 3 and 5 cm working distances and Gen 3 versus Gen 4 at all working distances, which are not significant.

Images of the resolution target were acquired with both the second-, third-, and fourth-generation KeyScopes and a SOC laparoscope at 3 to 13 cm from the target. The smallest resolvable feature was quantified in Fig. 3 where a lower value indicates superior resolution. As seen, the second-generation KeyScope, which had a 0.3 MP CMOS camera, achieved comparable resolution to the SOC laparoscope. The third- and fourth-generation KeyScopes, which have a 2.0 MP CMOS camera, further improved the resolution and consistently displayed finer resolution (86.46  μm) compared with the SOC laparoscope (108.93  μm).

**Fig. 4:**
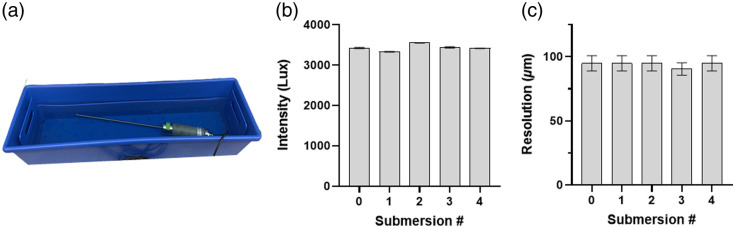
Waterproof testing. (a) Experimental set-up for submersion of the KeyScope in Cidex. (b) KeyScope LED light intensity was measured after each repeated submersion of the second-generation KeyScope. (c) KeyScope resolution measured after each repeat submersion of the second-generation KeyScope. Error bars indicate standard deviation, and each group had a sample size of n=5. No significant differences were observed between submersions.

To confirm that the revised form factor was waterproof, the entire KeyScope was submerged in Cidex for 30 min, rinsed with distilled water for 30 s, then allowed to air dry (n=4) as illustrated in Fig. 4(a). No signs of water ingress were observed, and the LEDs and cameras maintained their full functionality after each submersion [Figs. 4(b) and 4(c)].

### 3.2 Light Intensity Testing and Manufacturability Assessment

**Fig. 5:**
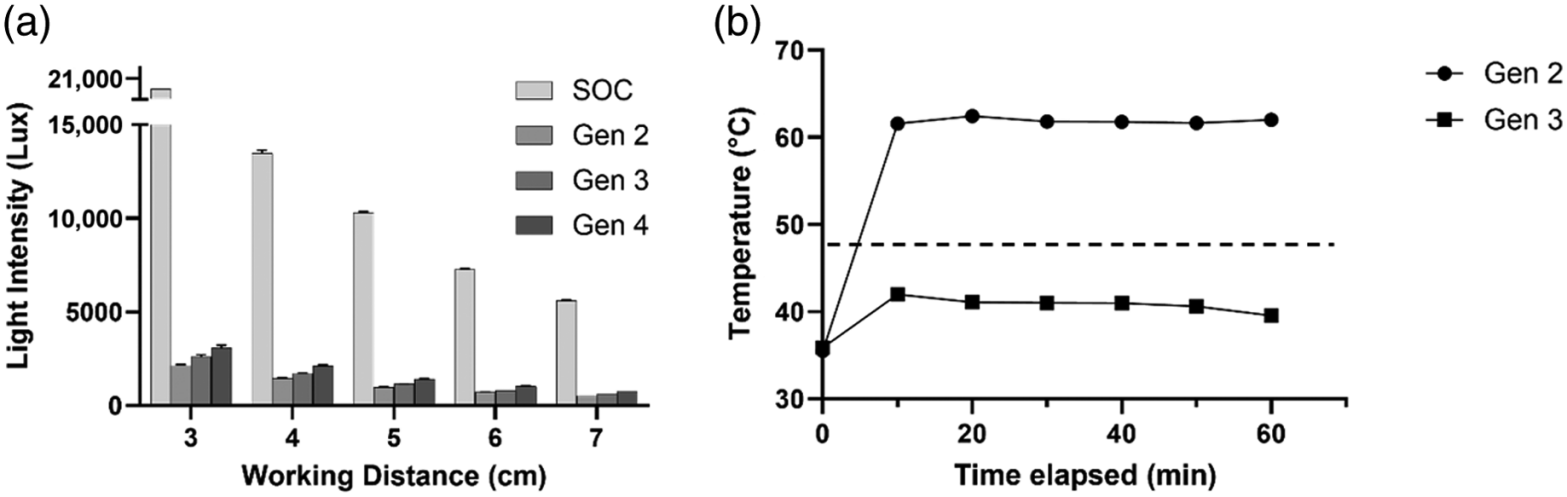
Light output and heat generation. (a) Illumination testing comparing the second- and third-generation KeyScope to a SOC laparoscope set at 100% intensity. The second- and third-generation KeyScope have decreased light intensity compared with the SOC. At the 3 cm working distance, the SOC laparoscope lux exceeded the luxmeter detection limit of 20,000 lux, which is the data point presented on the graph. Error bars indicate standard deviation, and all groups had a sample size of n=5. (b) Thermal testing of the tip of the second- and third-generation KeyScope. KeyScope temperature as a function of time was measured inside a 37°C humid beaker to mimic *in vivo* conditions, with the camera and LEDs on. The third-generation KeyScope operating temperature is ∼20°C lower than the second-generation KeyScope. The dotted line at 48°C represents the IEC 60601 temperature-approved limit for direct contact with human skin lasting less than 10 min in duration.

The light intensity was measured across the second-, third-, and fourth-generation KeyScopes at a variety of working distances and compared with a SOC laparoscope [Fig. 5(a)]. The third- and fourth-generation KeyScopes exhibited an increase of 500 and 900 lux, respectively, at a working distance of 3 cm compared with the second-generation KeyScope. *In vivo* testing was performed to confirm that this increase in light intensity was sufficient for laparoscopic surgery (Fig. 9).

Thermal testing was conducted to assess how pulsing the LEDs impacted heat generation at the tip of the KeyScope probe. As seen in Fig. 5(b), the third-generation KeyScope was 32% cooler than the second-generation KeyScope due to the pulsed LEDs. All temperatures observed with the third-generation KeyScope remained below the 48°C IEC 60601 approved temperature limit.

Third-generation KeyScopes were assembled by our engineering teams at the University of Maryland and Makerere University in Uganda to assess assembly time and to identify areas for improvement. Table 1 summarizes the average time for the four primary steps. The third step, circuit board assembly, took the longest, particularly at Makerere where the team did not have a fine soldering tip available, which made soldering the thin camera wires to the PCB challenging. Thus, the camera module and PCB were modified to have a male and female connector, respectively (generation 3.2, Fig. 2), which decreased the assembly time in Uganda by half to under an hour.

**Table 1:** Average assembly time for third-generation KeyScopes.

Step	Generation 3		Generation 3.2	
	UMD (n=1)	Makerere (n=3)	UMD (n=4)	Makerere (n=5)
1. Probe and top cap assembly	0:11:41	0:18:14	0:07:23	0:17:11
2. Handle assembly	0:20:51	0:11:58	0:01:07	0:04:06
3. Circuit board assembly	0:36:07	1:14:18	0:09:11	0:25:12
4. USB cord wiring + final closure	0:13:12	0:14:45	0:05:46	0:04:52
Total time (h:min:s)	1:21:51	1:59:15	0:23:27	0:51:21

### 3.3 Cautery Testing

**Fig. 6:**
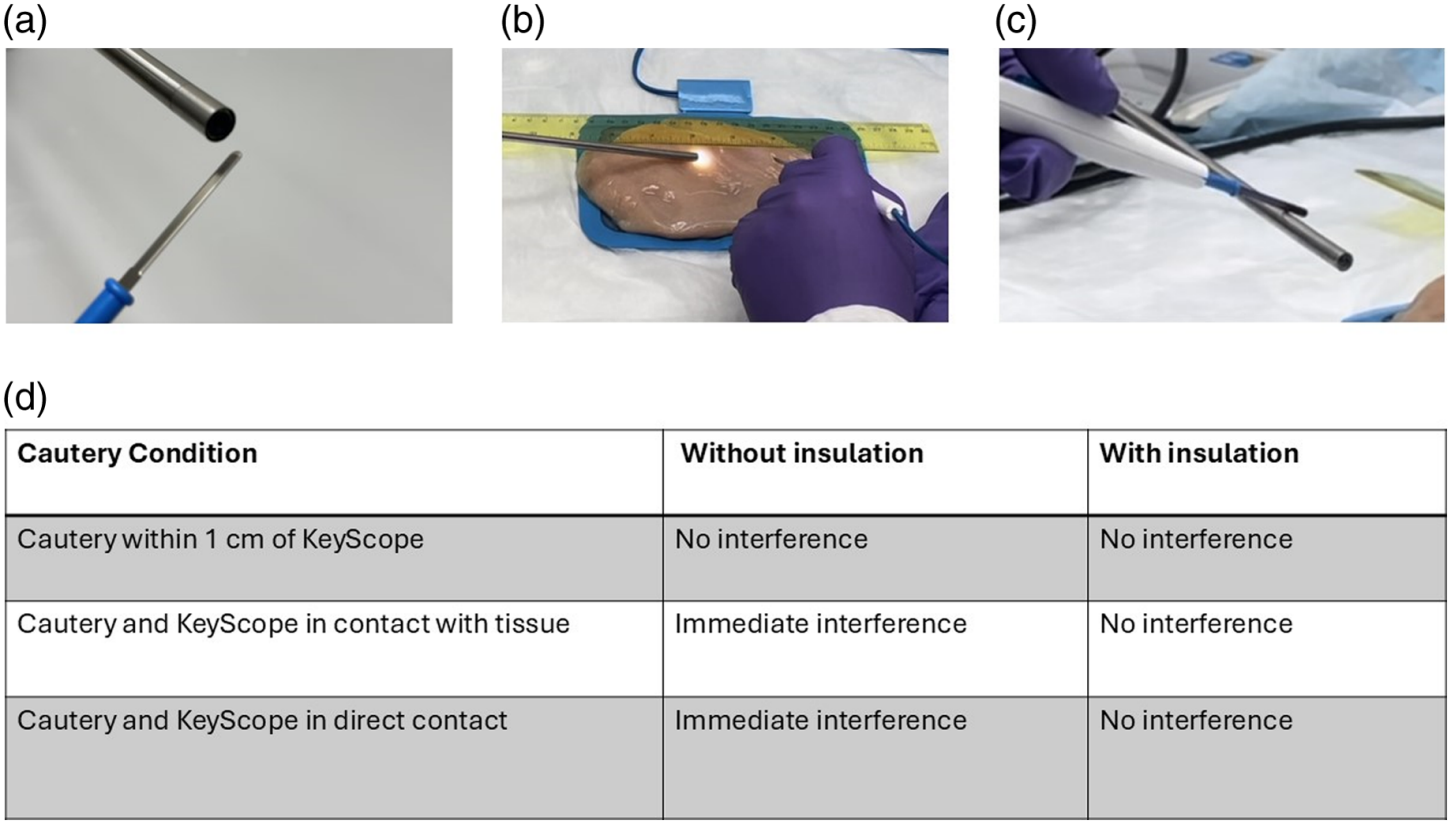
Cautery testing of the fourth-generation KeyScope. (a) The setup of the cautery testing when the cautery pencil is within 1 cm of the KeyScope. (b) The setup of the cautery testing when the cautery pencil and KeyScope are in contact with the tissue simultaneously. (c) The setup of the cautery testing when the cautery pencil and KeyScope are in direct contact. (d) Table of the effect on the KeyScope video feed based on the cautery conditions with and without exterior protective insulation.

To assess if there was any interference from cautery: (1) the KeyScope was brought near the cautery tool, (2) the KeyScope and the cautery tool were brought into contact with tissue, and (3) the cautery tool directly touched the KeyScope as illustrated in Figs. 6(a)–6(c). Interference was observed in cases 2 and 3 but not in case 1 [Fig. 6(d)]. Temporary insulative shielding was added to the third-generation KeyScope housing, which eliminated interference in all cases, confirming that a short circuit between the power ground and chassis ground was directing energy back into the camera circuitry and causing interference. In the fourth-generation KeyScope, the internal camera, and LED ground wires were electrically isolated from the housing ground. As seen in Fig. 6(d), this eliminated all interference from cautery.

### 3.4 Image Quality Comparison

**Fig. 7:**
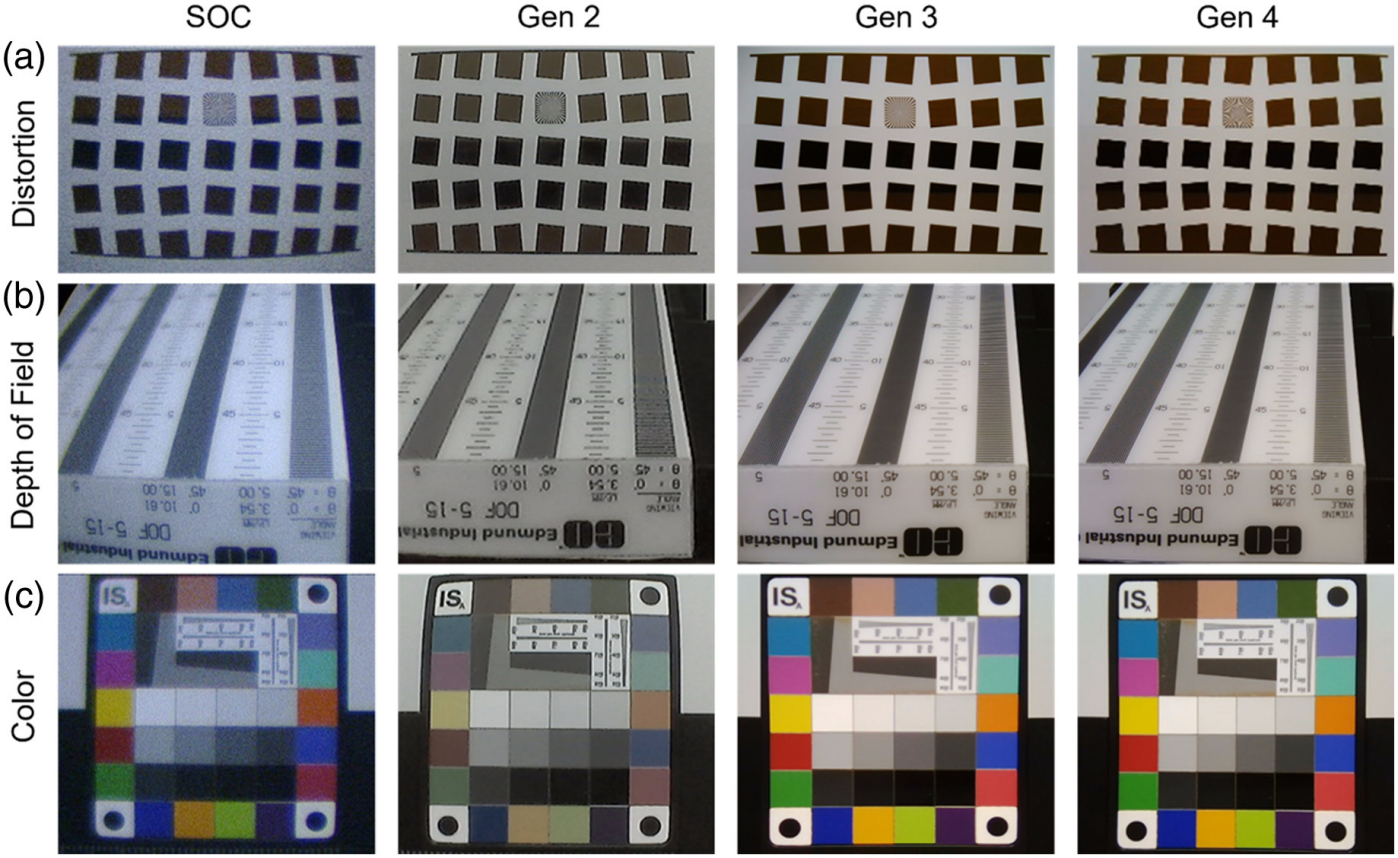
Target testing. Representative images captured with the SOC, second-, third-, and fourth-generation (gen) KeyScopes of (a) SFRplus distortion target, (b) 5 to 15 depth of field target, and (c) color checker target. All tests were conducted under the same lighting conditions in the PTC.

**Fig. 8:**
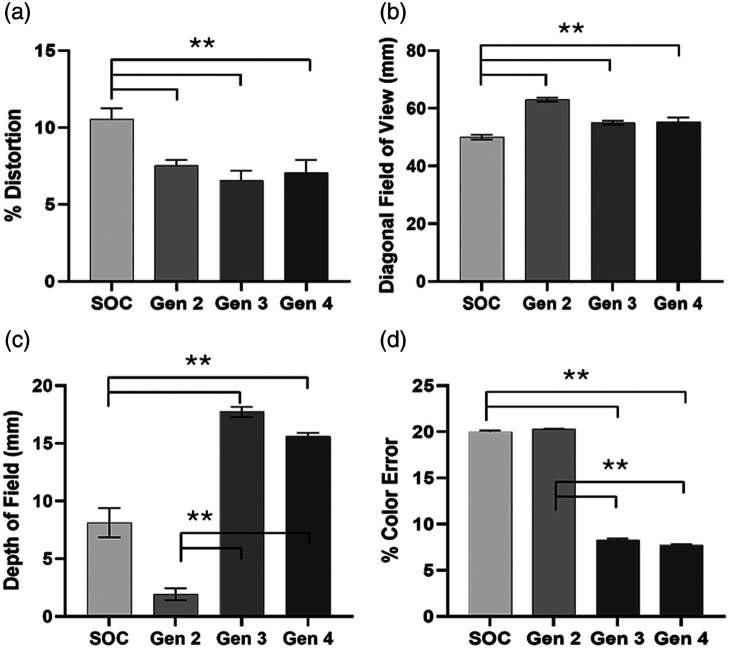
Image quality analysis. (a) Barrel distortion (% SMIA TV distortion) and (b) diagonal field of view were assessed using the SFRplus target and Imatest software. All KeyScope generations exhibit a lower % distortion than SOC, with a p<0.005. All KeyScope generations showed a larger field of view compared with the SOC laparoscope, with a p<0.005. (c) Depth of field was assessed using a 5 to 15 depth of field target. The furthest resolvable line pairs were determined using ImageJ software. Third- and fourth-generations have a significantly larger depth of field compared with SOC and second-generation with a p<0.001. (d) Color accuracy was assessed using the color checker target and the percent error of $\Delta C_{\mathrm{ab}}$ was quantified using Imatest software. The third- and fourth-generation KeyScopes displayed improved color accuracy compared to SOC and second-generation, with a p<0.001. Error bars indicate standard deviation, and each group had a sample size of n=5.

To ensure that image quality was maintained across generations of the KeyScope, the distortion, field of view, color accuracy, and depth of field were quantified and compared to a SOC laparoscope (Figs. 7 and 8). All measures were consistent or improved across generations of the KeyScope. All KeyScope generations exhibit a lower percent distortion than the SOC laparoscope (p<0.005). For example, the fourth-generation KeyScope obtained minimal distortion at 3 cm of <7.05%, which is significantly lower than the SOC laparoscope (<10.54%). Similarly, all KeyScope generations showed a larger field of view compared to the SOC laparoscope (p<0.005). The diagonal field of view for the fourth-generation KeyScope (5.52 cm) was slightly larger than the SOC (5.0 cm) at the same 3 cm working distance, enabling surgeons to see a slightly larger field of view during surgery. The third- and fourth-generation KeyScopes improved color accuracy compared to the second-generation KeyScope and SOC laparoscope (p<0.001). The fourth-generation KeyScope yielded less color error (<7.76%) than the SOC (<20.01%) and therefore more accurately reflects the colors of anatomical structures within the abdomen. Finally, the third- and fourth-generation KeyScopes have a significantly larger depth of field compared to the second-generation KeyScope and SOC laparoscope (p<0.001). Specifically, the depth of field was larger for the fourth-generation KeyScope (15.6 mm) compared with the SOC laparoscope (8.12 mm) at a 3 cm working distance, which is advantageous for keeping the entire surgical field in focus.

**Fig. 9:**
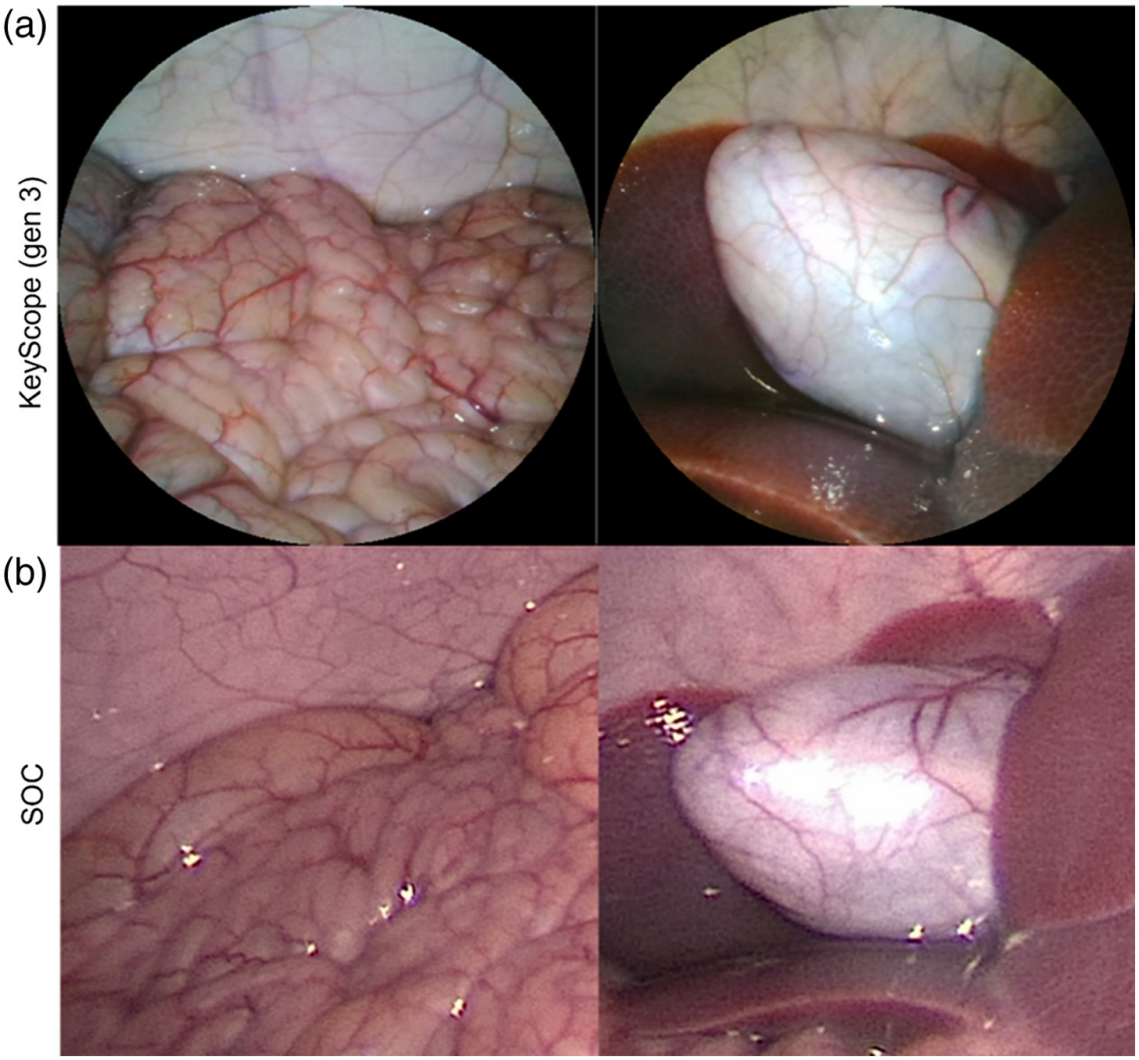
Pictures of porcine anatomy captured with the third-generation KeyScope (a). Similar fields of view were captured with the SOC laparoscope (b). The first column shows the intestines and abdominal wall, whereas the second column shows the gallbladder and liver.

A pilot *in vivo* study with the second-generation KeyScope revealed the current LEDs only illuminated the foreground anatomy, leaving the background of the cavity dark (Fig. S1 in the Supplementary Material). Thus, the pulsed LED feature was incorporated into the third-generation KeyScope, which was tested in the porcine model, and pictures were taken with the KeyScope and SOC laparoscope to enable side-by-side comparison (Fig. 9). As seen, similar anatomical features and details can be discerned both in the foreground as well as the background, suggesting that the KeyScope can provide comparable image quality *in vivo*, and can fully illuminate the abdominal cavity.

## 4 Discussion

While the initial version of the low-cost laparoscope demonstrated the feasibility of eliminating the laparoscopic tower and fiber optics and replacing them with consumer-grade electronics, further areas of improvement were needed. Specifically, the color accuracy, distortion, and field of view were initially comparable with a SOC laparoscope; however, the resolution and depth of field were comparable, though only at close working distances (3 to 4 cm away from the target). Further, the temperature of the initial design stayed significantly cooler (48°C) compared with SOC laparoscopes, which can reach temperatures of 100°C.^19^ However, the range of optimal focal distances was initially too limited (which impacted the resolution and depth of field at longer working distances) and the lux output was insufficient for laparoscopic surgery. These limitations were addressed through an iterative human-centered design approach.

First, the range of optimal focal distances was increased by changing the camera and the lens system coupled to the camera within the KeyScope. This addressed several challenges, including significantly improving the resolution of the KeyScope at longer working distances. With the second-generation KeyScope, comparable resolution to the SOC laparoscope was achieved across the range of working distances commonly used in laparoscopic surgery (3 to 13 cm). Even larger improvements in resolution were observed when the number of pixels was increased from 0.3 to 2.0 MP in the third-generation KeyScope, which achieved superior resolution at all working distances compared with the SOC laparoscope. The updated lens system also increased the depth of field of the third-generation KeyScope to 17 mm, beyond that of the SOC laparoscope (8 mm), allowing for more of the three-dimensional tissue topography within the abdomen to simultaneously be in focus within the KeyScope field of view.

The second significant improvement to the KeyScope was the increased light output from the LEDs to sufficiently illuminate the abdominal cavity. The amount of light required for sufficient illumination of the abdominal cavity with the KeyScope system was previously unknown because the KeyScope camera is more sensitive than the SOC camera and does not have any coupling to fiber optics, where a significant amount of signal can be lost in SOC laparoscopes. The KeyScope camera is placed at the front of the probe; consequently, less light is required to obtain a crisp image. Thus, a pilot *in vivo* assessment was performed. Initial *in vivo* testing indicated that insufficient illumination was achieved with the second-generation KeyScope, as the background anatomy was dark due to lack of illumination. Thus, in the third-generation KeyScope, brighter pulses of light were used to illuminate the abdominal cavity to increase the illumination by ∼300  lux (at 3 cm working distance) without overheating the camera. Pulsing the light in the third-generation KeyScope had the additional benefit of decreasing the heat generated at the tip of the probe from ∼60°C down to ∼40°C. This is significantly cooler than the SOC operating temperatures, which can reach 100°C,^19^ and is also below the IEC 60601 temperature approved limit (<48°C) for direct contact with human skin lasting less than 10 min in duration.^20^ Thus, the KeyScope has the added safety advantage of avoiding operating room fires or inadvertent intestinal burns—complications that are well-documented with SOC laparoscopes.^21^ Importantly, pilot *in vivo* testing indicated that sufficient illumination was achieved using the pulsed LED approach in the third-generation KeyScope, as all anatomy in the FOV were illuminated and visible. Larger animal studies in which surgeons performed a variety of tasks with our latest KeyScopes have been performed.^22^

Using a human-centered design approach with feedback from surgeons and engineers in Uganda, important improvements needed were identified. The first key feature was to design the KeyScope so that it could be easily reprocessed for the next patient by locally available methods. Although laparoscopic equipment in high-income countries is typically sterilized via autoclave or ethylene oxide gas, these methods are often unavailable in LMICs due to high operational and maintenance costs.^7^ SOC steam and gas sterilization methods can cost approximately $130,000 USD with an estimated $19,000 USD annual cost of repairs.^23^ Rather, chemical submersion is commonly used for low-cost reprocessing, with a comparative annual cost of $10,000 USD.^8^^,^^24^ However, to withstand submersion, the entire KeyScope needed to be waterproof. Thus, in the second-generation KeyScope, several waterproofing strategies were implemented, which included a series of medical-grade epoxy, O-rings, and threaded components that tightly fit together. Repeated submersion could be performed without any water ingress or impact on functionality, suggesting our new design is watertight. Lifetime testing in which hundreds of repeated submersions are performed is currently underway to ensure waterproofing and durability. Although the KeyScope contains the CMOS detector which is a fragile component, this technology is contained within the first centimeter of the probe tip. The remainder of the components are durable insulated electrical wires within the probe, handle components, and USB cords. Given that the KeyScope was designed for manufacturing in Uganda, the technology can be more easily repaired by local engineers and technicians when damage is sustained. Conversely, SOC laparoscopic probes consist of delicate rod lens systems, and the fiber optic cables that connect to the laparoscopic tower can break when bent, decreasing light output.^25^

The second important design element was to consider efficient manufacturing in Uganda. Thus, pilot manufacturing runs of several units were performed in the United States and in Uganda to compare build times and assess the feasibility of doing final assembly in Uganda. The pilot build in Uganda took on average ∼38  min longer than in the United States. A large majority of this difference was due to a single step—final PCB assembly—during which the camera wires were soldered directly into a 2×4 array on our custom PCB (Table 1). Because the Ugandan team did not have fine soldering equipment, this step was particularly challenging and could lead to a high scrap rate. Thus, in the next iteration, the fine soldering step was eliminated by designing a male and female connector that came pre-assembled in the camera module and custom PCB, respectively. As seen, this decreased build time in the United States by a factor of 4 down to less than 25 min and decreased the time by a factor of 2 down to under an hour in Uganda. These key features—waterproofing and designing for local manufacturing—would not be possible without key partnerships with surgeons and engineers in an LMIC.

The need for another essential design improvement was discovered during the pilot *in vivo* testing with cautery. Subsequent bench testing with cautery demonstrated that electrical interference led to the camera disconnecting approximately 1% of the time. This loss of the video feed could potentially lead to an adverse surgical outcome; thus, our fourth-generation KeyScope focused on eliminating this error by separating the housing (chassis) ground from the camera ground. This crucial feature highlights the importance of *in vivo* testing before delivering a product, increasing the likelihood that it will ultimately work in its intended environment.

*In vivo* testing in a porcine model has been completed and reported in a separate study.^22^ Our team is currently seeking regulatory approval through the National Drug Authority (NDA) in Uganda, after which the KeyScope will be tested in a first-in-human clinical trial at the Uganda Cancer Institute to obtain cancer biopsies. Simultaneously, we are working with a medical device manufacturing company in Uganda with the goal of local manufacturing, followed by local, regional, and worldwide distribution to other LMICs. Finally, we are developing additional features for the KeyScope, including the addition of a 30-deg angle to the tip of the scope to improve intraoperative exposure.

## 5 Conclusion

The performance of the KeyScope was improved using human-centered design by engaging surgeons and engineers in Uganda and the United States. Through each iteration, performance specifications were maintained while improving key features. These new features included improving the resolution of the device at longer working distances, waterproofing the entire device, increasing the brightness of the LEDs while simultaneously decreasing the temperature profile, and eliminating interference from cautery. Additional design modifications improved the efficiency of manufacturing locally in Uganda. The KeyScope has been uniquely designed to enable laparoscopic surgery in LMICs, which could have beneficial outcomes for many patients.

## Supplementary Material

10.1117/1.BIOS.2.2.022302.s01

## Data Availability

The data generated in this study are available through GitHub: https://github.com/abarnes9/BIOS-Improved-KeyScope-performance-.
