# Supplementary material for: Improved performance and design of a low-cost laparoscope to enable laparoscopic surgery in low-income countries
Source: Biophotonics Discov. 2025 Feb 3;2(2):022302. doi: 10.1117/1.BIOS.2.2.022302 (PMC13098739; doi:10.1117/1.BIOS.2.2.022302)
Supplement: Supplementary file 1 [file BIOS_002_022302_SD001.pdf]

Supplementary Material

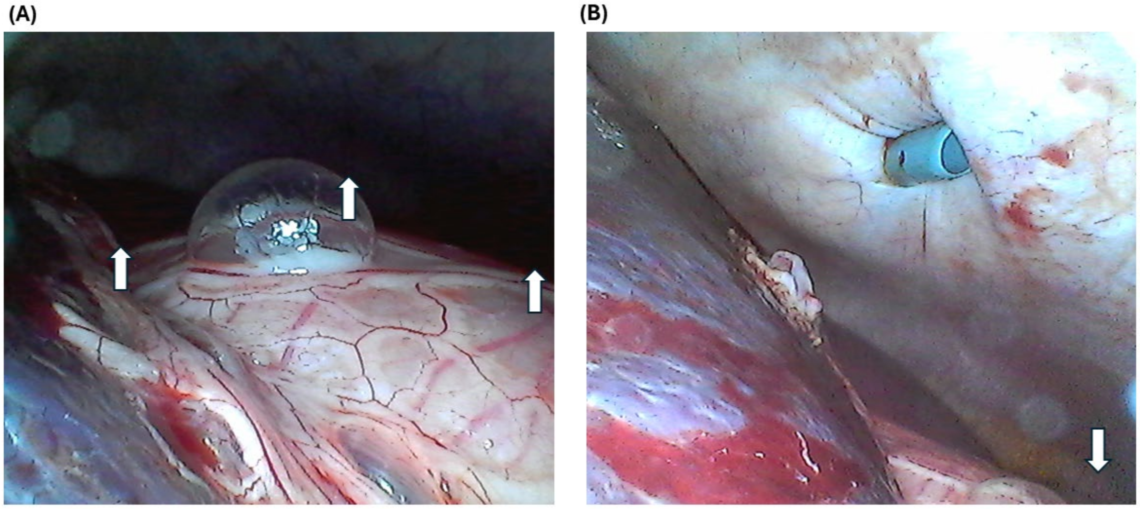

Fig. S1. Pictures of porcine anatomy captured with the second-generation KeyScope. White arrows point to dimly lit background, revealing the need to increase LED brightness for complete cavity illumination. (A) Image of the porcine stomach taken with the second generation KeyScope. (B) Image of the porcine abdominal wall taken with the second generation KeyScope.
